# Genetic diversity in the block 2 region of the merozoite surface protein-1 of *Plasmodium falciparum *in central India

**DOI:** 10.1186/1475-2875-11-78

**Published:** 2012-03-22

**Authors:** Praveen K Bharti, Man M Shukla, Yagya D Sharma, Neeru Singh

**Affiliations:** 1 Regional Medical Research Centre for Tribals, Nagpur Road, Garha, Jabalpur 482003, Madhya Pradesh, India; 2 National Institute of Malaria Research, Field Station, Jabalpur, Madhya Pradesh, India; 3 Department of Biotechnology, All India Institute of Medical Sciences, New Delhi, India

## Abstract

**Background:**

Malaria continues to be a significant health problem in India. Several of the intended *Plasmodium falciparum *vaccine candidate antigens are highly polymorphic. The genetic diversity of *P. falciparum *merozoite surface protein-1 (MSP-1) has been extensively studied from various parts of the world. However, limited data are available from India. The aim of the present study was a molecular characterization of block 2 region of MSP-1 gene from the tribal-dominated, forested region of Madhya Pradesh.

**Methods:**

DNA sequencing analysis was carried out in 71 field isolates collected between July 2005 to November 2005 and in 98 field isolates collected from July 2009 to December 2009. Alleles identified by DNA sequencing were aligned with the strain 3D7 and polymorphism analysis was done by using Edit Sequence tool (DNASTAR).

**Results:**

The malaria positivity was 26% in 2005, which rose to 29% in 2009 and *P. falciparum *prevalence was also increased from 72% in 2005 to 81% in 2009. The overall allelic prevalence was higher in K1 (51%) followed by MAD20 (28%) and RO33 (21%) in 2005 while in 2009, RO33 was highest (40%) followed by K1 (36%) and MAD20 (24%).

**Conclusions:**

The present study reports extensive genetic variations and dynamic evolution of block 2 region of MSP-1 in central India. Characterization of antigenic diversity in vaccine candidate antigens are valuable for future vaccine trials as well as understanding the population dynamics of *P. falciparum *parasites in this area.

## Background

Madhya Pradesh (MP) is situated in the central part of India, and is a highly malarious state contributing 9% of all malaria cases in the country [1]. *Plasmodium falciparum *infection has dramatically increased in MP in recent years and is associated with life-threatening complications in both children and adults [2,3].

The merozoite surface protein-1 (MSP-1) is a leading vaccine candidate antigen. It is the most abundant surface protein on the blood stage of *P. falciparum*, and it is thought to play a role in erythrocyte invasion [4]. The primary structure of MSP-1 is polymorphic and 40% of the amino acid residues are different in different allelic forms in *P*. falciparum [5]. The precursor of MSP-1 is a protein comprising 1,720 amino acids, including a 20-amino-acid signal sequence (SS) and a signal for anchoring the protein at the cellular surface via a GPI moiety (GA). MSP-1 divided into 17 blocks, which were either variable, conserved or semi-conserved [6,7]. Sequences of blocks 1, 3, 5, 12 and 17^th ^are conserved, and blocks 2, 4, 6, 8, 10, 14 and 16 diverge extensively while in the remaining blocks 7, 9 11, 13 and 15 are semi-conserved. Variations in the sequences are dimorphic in nature with the exception of polymorphic tripeptide encoding region in block 2.

The block 2 region includes three allele families: K1, MAD20, and RO33. Alleles in K1 and MAD20 contain antigenically unique, tripeptide repeats, with extensive diversity in the number of repeats [7]. RO33 lacks the tripeptide repeats observed in the other two families; however, outside block 2, this allele is similar to the MAD20 type [8]. Fragment size in the three block 2 allele families has commonly been used as a molecular marker in studies of malaria transmission dynamics and host immunity in *P. falciparum *malaria [9-13]. The protective immune responses have also been observed against the motifs present in the major allele families of block 2 and while the evidence suggests that the allele families are maintained by selection, it is not clear how selection operates against the number of tandem repeats [14-16].

The purpose of this study was to explore the extent of genetic variation in MSP-1 block 2 over the years in central India for studying as a molecular marker in epidemiologic investigations, malaria transmission dynamics and finally help in vaccine design under selection pressure.

## Methods

### Study sites

**Figure 1:**
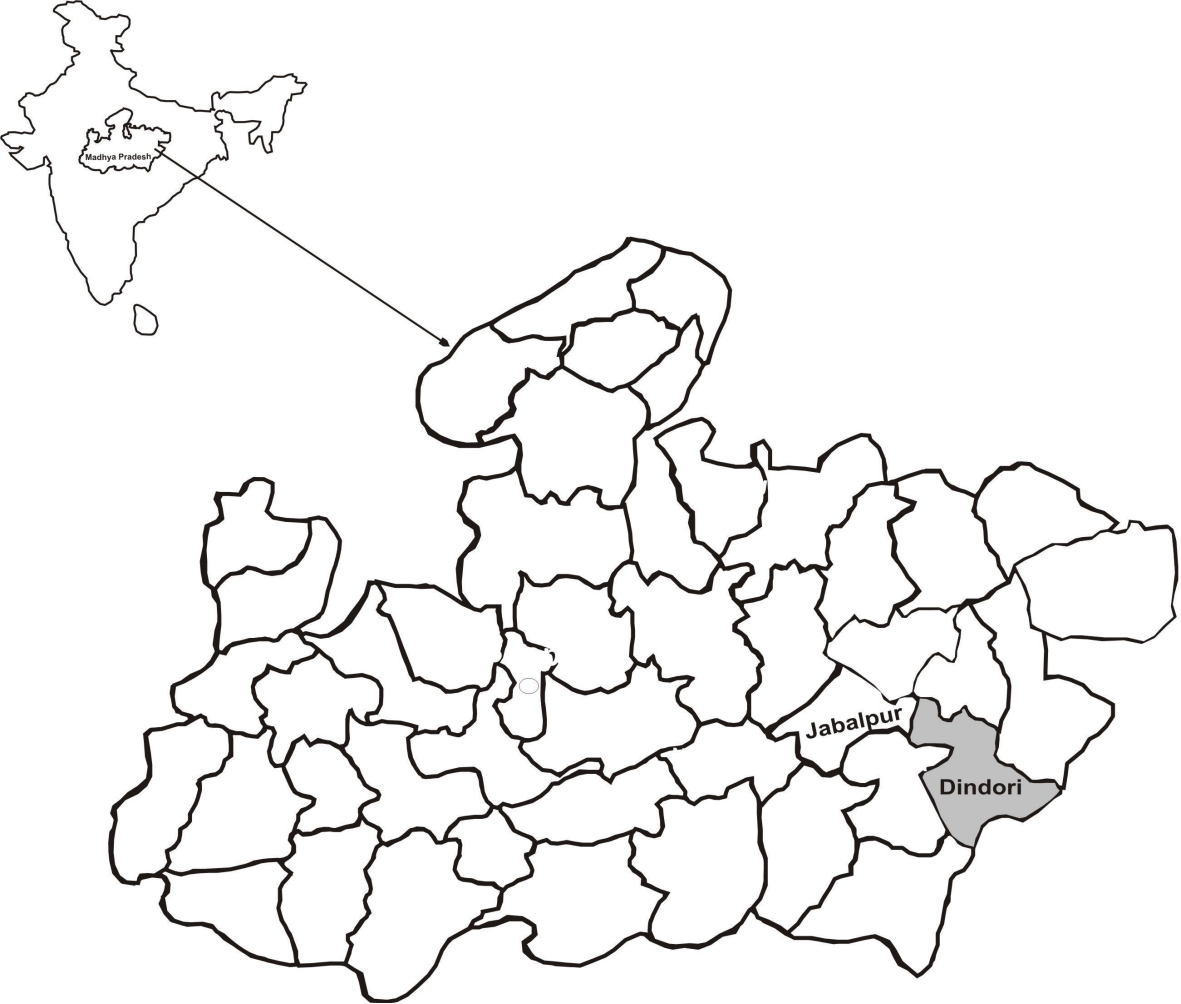
**Map showing district Dindori, Madhya Pradesh, Central India**.

The present study was carried out in Baigachak area of Dindori district, Madhya Pradesh, India (Figure 1), from July 2005 to November 2005 and July 2009 to December 2009 during peak transmission season. It is a highly malarious district in the State of Madhya Pradesh with a very high transmission rate [17]. Patients ranging between one and 59 years of age presenting with fever and symptoms of *P. falciparum *malaria were screened for malaria parasites after obtaining consent. Fever history was obtained from the patient or by an accompanying person (in the case of children). Physical examination of the patients was performed and axillary temperature recorded.

### Sample collection

Parasitologic surveys were carried out to collect blood smears from all fever cases and cases with history of fever. Blood smears were stained with Jaswant Singh, and Bhattacharji (JSB) stain examined under light microscope for *Plasmodium *species identification [18].

Three to five drops of finger prick blood was blotted on 3 MM filter paper (Whatman) to study genetic diversity of MSP-1. Consent from the study subjects was taken before collection the blood samples.

### DNA isolation from filter paper

Blood spotted area was punched and put into a 1.5 ml tube. The blood spot were soaked in 150 μl TE buffer (10 mM Tris, 0.1 mM EDTA, pH 8.0) and incubated for an hour at RT. After one hour incubation, tubes were placed in dry bath at 50°C and incubated for 15 minutes and punched by pipettes tips several times. Finally the tubes were incubated at 97°C for 15 minutes and centrifuged at 8,000 rpm for 2 minutes. Supernatant was aspirated and stored at -20°C for PCR amplification.

### PCR amplification of the *msp1 *gene

The primary PCR was set up for the amplification of block 2 region by using the primers MSP1A (forward): 5'-CACAATGTGTAACACATGAAAG-3' and MSP1B (reverse): 5'- AGTACGTCTAATTCATTTGCAC -3'. The 646 bp primary PCR product was diluted 1:10 and was used for the nested PCR. A nested PCR of a 555 bp product was amplified by using the primers MSP1C(forward): 5' -TAGAAGCTTTAGAAGATGCAG-3' and MSP1 D(reverse): 5' GACAATAATCATTAGCACATAC 3'and sequenced. The primary PCR was performed in a volume of 20 μL with 0.175 U of *Taq *DNA polymerase, 0.2 mM each dNTP, 0.4 μM each primer, and 1 mM MgCl_2_. The reaction was allowed to proceed for 35 cycles after an initial denaturation at 94 C for 1 minute, annealing at 55 C for 1 minute, and extension at 72 C for 1 minute. Final extension was at 72 C for 10 minutes. The nested PCR was performed with annealing at 53 C for 25 cycles. Other nested PCR conditions were the same as those described for the primary PCR [17]. The PCR products were resolved on a 2% agarose gel.

### Nucleotide sequencing

The PCR products were purified from the agarose gel by using HyYeld™ gel/PCR DNA extraction kit (Real Biotech Corp., Teipei Country, Taiwan), as per the manufacturer's recommended protocol. From 200 to 250 ng of the gel-purified product was used with the ABI Big Dye Terminator Ready Reaction Kit Version 3.1 (PE Applied Biosystems Foster City, CA 94404, USA) for the sequencing PCR. The sequencing PCR was performed in a volume of 20 μL with 1 μL to Terminator Ready Reaction Mix (TRR), 3.2 pmol of gene specific primer MSP1C (555 bp of block 2 region) and 0.5X sequencing buffer. Cycling conditions for the sequencing PCR included 25 cycles of denaturation at 96 C for 10 seconds, annealing at 50 C for 5 seconds, and extension at 60°CC for 4 minutes. Templates were purified and sequenced on an ABI Prism 310 Genetic Analyzer (PE Applied Biosystems).

### Sequence analysis

Sequence obtained was translated using the Edit Sequence tool (DNASTAR). The translated sequences were then aligned using the MEGALIGN program (DNASTAR, INC., Madison, WI). Nucleotide sequences are submitted to the GenBank database.

The expected heterozygosity was calculated by use of the formula ${\text{H}}_{\text{E}}=\left[\text{n}/\left(\text{n}-\text{1}\right)\right]\times \left[\left(\text{1}-\Sigma \text{p}{\text{i}}^{\text{2}}\right)\right]$, where n is the number of samples and pi the frequency of allele i. H_E _is the probability that two alleles randomly drawn from the population sample are different. The mean multiplicity of infection (MOI) was calculated as the total number of clones divided by the number of positive samples for marker gene. Allele frequencies were further compared between two year populations and P value was calculated for significance.

### Ethical approval

The study was approved by the Scientific Advisory Committee, Ethical Committee of Regional Medical Research Centre for Tribals, Jabalpur, MP, India and informed consent and human subjects guidelines were followed.

## Results

The overall malaria positivity was 26% in 2005, which rose to 29% in 2009 (Table 1) among the symptomatic individuals. The *P. falciparum *prevalence was increased from 72% in 2005 to 81% in 2009 {Odd ratio (OR) = 1.33 (1.0-1.77) and *p *< 0.05}.

**Table 1:** Malaria cases in study area with different age groups among symptomatic individuals

Study Year	Age groups	No. of Patients Screened	No. of Positive Cases	Pf	Pv	Mixed (Pf + PV)	No. of Sample sequenced
**2005**	< 5 yr	128	31	23	6	2	16
	
	5-15 yr	356	89	65	21	3	41
	
	> 15 yr	169	49	33	13	3	14

**2009**	< 5 yr	118	40	30	8	2	23
	
	5-15 yr	238	64	51	10	3	39
	
	> 15 yr	221	61	53	7	1	36

**Total**		**1230**	**334**	**255**	**65**	**14**	**169**

Pf Plasmodium falciparum, Pv Plasmodium vivax

### Sequence polymorphism in block-2 of *msp1*

The 555 bp polymorphic region of block 2, merozoite surface protein 1 gene was amplified. A total of 169 *P. falciparum *infected blood samples from 2005 and 165 samples from 2009 were used for the amplification and sequencing of merozoite surface protein 1, block 2. The amplified fragments of 169 (71 from 2005, 98 from 2009) samples were sequenced and their sequences were analysed with three alleles, K1, MAD20 and RO33. Comparison of the sequences showed that all these isolates belong to one of these three alleles. The overall allelic prevalence was recorded which was higher in K1 (51%) followed by MAD20 (28%) and RO33 (21%) in 2005 while in 2009 RO33 was highest (40%) followed by K1 (36%) and MAD20 (24%).

**Figure 2:**
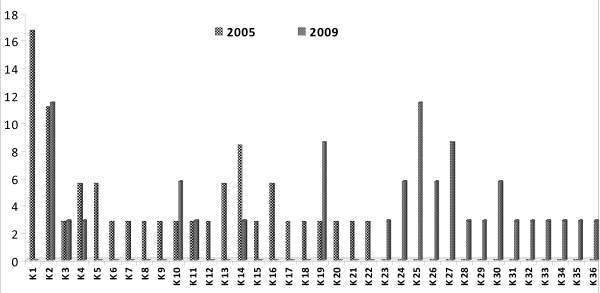
**Temporal variation in frequency distribution of K1 allelic family of *Plasmodium falciparum *msp1 haplotypes between 2005 and 2009 from study area**. Frequencies are shown on the vertical axis and number of variants on X axis.

**Figure 3:**
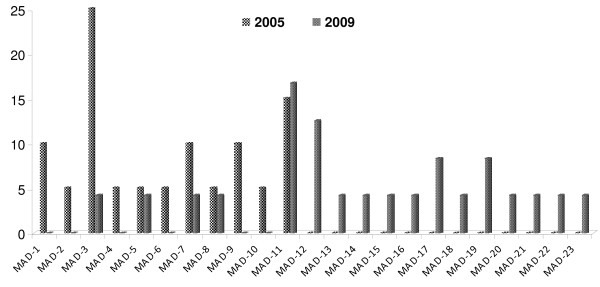
**Temporal variation in frequency distribution of MAD20 allelic family of *Plasmodium falciparum *msp1 haplotypes between 2005 and 2009 from study area**. Frequencies are shown on the vertical axis and number of variants on X axis.

**Figure 4:**
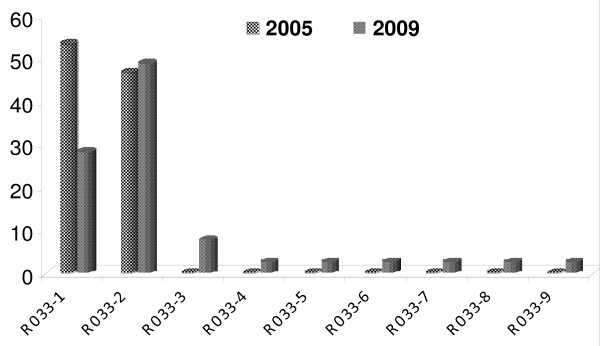
**Temporal variation in frequency distribution of RO33 allelic family of *Plasmodium falciparum *msp1 haplotypes between 2005 and 2009 from study area**. Frequencies are shown on the vertical axis and number of variants on X axis.

In the block 2 of MSP1, the nucleotide and the deduced amino acid sequence were found to be highly polymorphic among the isolates. All the nucleotide changes in these isolates were non-synonymous, as a result, the deduced amino acid variations corresponded to one or other allele. A total 22 types of variants were found in the K1 type alleles in 2005 and 21 types of variants in 2009(Additional file 1: Figure S1). Out of these 21 variants only seven belong to 2005 types and the remaining 14 were new variants (Figure 2). MAD20 type of allelic had limited 11 variants in 2005 while in 2009 total 17 variants were found (Additional file 1: Figure S2). Out of these 17 variants only five belong to 2005 types and the remaining 12 were new variants (Figure 3). The RO33 showed an almost semi-conserved pattern, showing only two variants in 2005 samples but in 2009 (Additional file 1: Figure S3) it showed nine variants (Figure 4).

Mean multiplicity of infection (MOI) was greater in 2009 than in 2005 for *msp1 *genes (*msp1*: 1.34 ± 0.44 vs. 1.27 ± 0.47; *p *> 0.05) Table 2. The frequencies of polymorphisms in polymorphic blocks 2, were compared from 2005 to 2009 (Table 2). A significantly frequency variation was observed in RO33 allelic family (GenBank database accession numbers JF460898, JF460899, JF460900, JF460901, JF460902, JF460903, JF460904, JF460905, JF460906, JF460907, JF460908, JF460909, JF460910, JF460911, JF460912, JF460913, JF460914, JF460915, JF460916, JF460917, JF460918, JF460919, JF460920, JF460921, JF460922, JF460923, JF460924, JF460925, JF460926, JF460927, JF460928, JF460929, JF460930, JF460931, JF460932, JF460933, JF460934, JF460935, JF460936, JF460937, JF460938).

**Table 2:** Genetic diversity of MSP1 from 2005 and 2009

	2005	2009	OR	p value
**No. of Samples**	71	98		

**No. of alleles**	35	47		

**No. of different MSP1- K1 alleles**	22	21	1.85 (0.95-3.62)	*p *> 0.05

**No. of different MSP1- MAD20 alleles**	11	17	1.21 (0.57-2.55)	*p *> 0.05

**No. of different MSP1- RO33 alleles**	2	9	0.40 (0.19-0.85)	*p *< 0.05

**No. of clones**	90	131		

**MOI**	1.27 ± 0.44	1.34 ± 0.47		*p *> 0.05

**H_E_**	0.94	0.91		

*MOI *mean multiplicity of infection

*H_E _*expected heterozygosity. This is defined as the probability that two randomly chosen alleles are different in the population

*OR *Odd ratio

## Discussion

The genetic diversity of *P. falciparum, msp1*genes was investigated from the field isolates of high transmission area over the five-year periods from central India. Of the 334 isolates, MSP1 sequencing was successfully completed in 169 isolates.

Most malaria vaccine-candidate antigens are highly polymorphic surface proteins that elicit variant specific immunity. Therefore, the evolutionary relationships could be explored for the design of vaccines based on ancestral sequences, with the potential for including cross-protection against a wide range of antigenic variants. Thus, the understanding of mechanisms and patterns of genetic recombination and sequence variation may help in designing a vaccine that represents the worldwide repertoire of polymorphic malaria surface antigens.

The MSP-1, with numerous alleles and differing in the length of the genes, have been extensively studied and their genetic polymorphisms were used to describe clonality of infections in a large number of studies. Length variability in MSP families is mainly results from repeat sequences. The alleles of MSP-1 belong to the allelic groups K1, MAD20 and RO33 with high variability when comparing the groups, but less variability within them. Minor amino acid diversity is created in malaria parasite antigens by single-nucleotide replacement. Dependent on the degree of amino acid substitution (highly variable, semi-conserved, conserved), MSP-1 has been categorized into 17 blocks [6]. Genetic recombinations account for most variation seen in malarial antigens, since it occurs in several orders of magnitude more frequently than mutation [19]. Differential prevalence of dimorphic MSP1 epitopes had previously been reported by Conway et al. from West Africa (Gambia and Nigeria) and Brazil (eastern Amazon) [20].

Block 2 is of particular interest, as it exhibits repetitive tri-nucleotides and appears to be subjected to rapid intragenic recombination process, comparable to those of the csp gene. It has been shown that IgG antibodies are important in acquired anti-malarial immunity against the most frequent subtypes of block 2 of MSP-1[15].

Previous studies of block 2 of MSP1 allelic types from India yielded information of varying patterns of diversity [21-23]. In the present study, results reveal that K1 alleles are dominant (51%) followed by MAD20 (28%) and rest were RO33 type alleles in 2005 samples, in contrast to a Colombia study in 1990, where only MAD20 and RO33 type alleles were reported and the K1 type alleles were missing. In Iran only limited numbers of K1 (7.6%) type alleles were reported by Mehrizi et al. [24]. Mahajan et al. reported all three types of alleles from India [21]. In the Zambian isolates of MSP1 from block2, 54% K types alleles, 35% RO33 and 11% of MAD20 like alleles were reported [5]. Analysis of 2009 samples in present study showed 36% K types alleles, 40% RO33 and 24% of MAD20-like alleles.

In another study carried out in Choea (north-west Colombia) in 1997, all three (MSP1 block 2) allelic types were detected although MAD20 was the predominant allele and K1 was less frequent [25]. The maximum variation in this study (22 variants in 2005 and 21 variants in 2009) was observed in the K1 allelic family. The extensive variation in the repeat region of block 2 was also reported by Tetteh et al. [5]. The highest variation was observed in the high transmission area of Orissa state, India by Ranjit and Sharma as compared to other states of India [22]. Simultaneously, a high degree of variation was recorded by Raj et al. in the high transmission area, as compared to the mesoendemic area in Orissa state [23]. In the present finding, RO33 type alleles were semi-conserved and only two variants were found in 2005 samples and increased to nine by 2009.

The level of antigenic diversity of *P. falciparum *populations in an area is likely to affect acquisition of immunity to malaria. Substantial variations in the prevalence of block 2 alleles during different study period indicates dynamic nature of *msp1 *genetic structure in *P. falciparum *populations. It is possible that acquisition of strain specific immunity may modulate the selection of different allelic variants and this may be one of the explanation for the observed findings. Sequence analysis of the present study identified numerous novel alleles and specific motif arrangements of *msp1*, block 2 allele sequences as reported by Escalante et al. [26]. Further genetic polymorphism appear to evolve faster in the higher transmission areas when compared to lower transmission areas [27,28]. The degree of polymorphism found in the present study is also consistent with the high level of transmission of malaria in the study area as reported previously by Singh et al., [29].

High level of MOI observed in this study fits with previous observations of an increased complexity of infection with increasing endemicity [30]. Over all findings from this study indicates dynamic evolution of variation in the *msp1 *gene of *P. falciparum *in the study area and it could serve as a good marker in studying the *P. falciparum *population in this region. In addition, extensive genetic variation in the block 2 region of MSP-1 makes it as useful genetic markers in differentiating parasite strains in clinical trials in this region.

## Conclusion

The present study reports extensive genetic variations and dynamic evolution of block 2 region of MSP-1 in Central India. Characterization of antigenic diversity in vaccine candidate antigens are valuable for future vaccine trials as well understanding the population dynamics of *P. falciparum *parasites in this area.

## Competing interests

The authors have no commercial or other association that might pose a competing interest.

## Authors' contributions

PKB carried out PCR amplification, DNA sequencing experiments and drafted the manuscript. MMS and PKB carried out patients' enrolments, collected clinical and epidemiological data and drafted the manuscript. YDS analysed sequencing data and drafted the manuscript. NS conceived and designed the study, monitor the field and laboratory experiments, analysed the data and drafted the manuscript. All authors read and approved the final manuscript.

## Supplementary Material

Additional file 1 **Figure S1**. Amino acid sequence alignment of the K1 allelic types of *Plasmodium falciparum msp1 *gene from central India. Figure S2. Amino acid sequence alignment of the MAD20 allelic types of *Plasmodium falciparum msp1 *gene from central India. Figure S3. Amino acid sequence alignment of the RO33 allelic types of *Plasmodium falciparum msp1 *gene from central India. Click here for file

## References

[B1] Singh N Dash AP Thimasarn K Fighting malaria in Madhya Pradesh (Central India): are we losing the battle? Malar J 2009 8 93 10.1186/1475-2875-8-93 19419588 PMC2687456

[B2] Singh N Nagpal AC Saxena A Singh MP Changing scenario of malaria in central India, the replacement of *Plasmodium viva *by *Plasmodium falciparu *(1986-2000) Trop Med Int Health 2004 9 364 371 10.1046/j.1365-3156.2003.01181.x 14996366

[B3] Jain V Nagpal AC Joel PK Shukla M Singh MP Gupta RB Dash AP Mishra SK Udhayakumar V Stiles JK Singh N Burden of cerebral malaria in central India (2004-2007) AmJTrop Med Hyg 2008 79 636 642 PMC2710578 18840756

[B4] Holder AA Blackman MJ Burghaus PA Chappel JA Ling IT McCallum-Deighton N Shai S A malaria merozoite surface protein (MSP1)-structure, processing and function Mem Inst Oswaldo Cruz 1992 87 37 42 1343716 10.1590/s0074-02761992000700004

[B5] Tetteh KK Cavanagh DR Corran P Musonda R McBride JS Conway DJ Extensive antigenic polymorphism within the repeat sequence of the *Plasmodium falciparu *merozoite surface protein 1 block 2 is incorporated in a minimal polyvalent immunogen Infect Immun 2005 73 5928 5935 10.1128/IAI.73.9.5928-5935.2005 16113313 PMC1231057

[B6] Tanabe K Mackay M Goman M Scaife JG Allelic dimorphism in a surface antigen gene of the malaria parasite *Plasmodium falciparu* J Mol Biol 1987 195 273 287 10.1016/0022-2836(87)90649-8 3079521

[B7] Miller LH Roberts T Shahabuddin M McCutchan TF Analysis of sequence diversity in the *Plasmodium falciparu *merozoite surface protein-1 (MSP-1) Mol Biochem Parasitol 1993 59 1 14 10.1016/0166-6851(93)90002-F 8515771

[B8] Hughes AL Positive selection and interallelic recombination at the merozoite surface antigen-1 (MSA-1) locus of *Plasmodium falciparum* Mol Biol Evol 1992 9 381 393 1584009 10.1093/oxfordjournals.molbev.a040730

[B9] Ariey F Chalvet W Hommel D Peneau C Hulin A Mercereau-Puijalon O Duchemin JB Sarthou JL Reynes JM Fandeur T *Plasmodium falciparum *parasites in French Guiana: limited genetic diversity and high selfing rate AmJTrop Med Hyg 1999 61 978 985 10.4269/ajtmh.1999.61.978 10674682

[B10] Da Silveira LA Dorta ML Kimura EA Katzin AM Kawamoto F Tanabe K Ferreira MU Allelic diversity and antibody recognition of *Plasmodium falciparum *merozoite surface protein 1 during hypoendemic malaria transmission in the Brazilian amazon region Infect Immun 1999 67 5906 5916 10531247 10.1128/iai.67.11.5906-5916.1999 PMC96973

[B11] Färnert A Rooth I Svensson, Snounou G, Björkman A: Complexity of *Plasmodium falciparum *infections is consistent over time and protects against clinical disease in Tanzanian children J Infect Dis 1999 179 989 995 10.1086/314652 10068596

[B12] Konaté L Zwetyenga J Rogier C Bischoff E Fontenille D Tall A Spiegel A Trape JF Mercereau-Puijalon O Variation of *Plasmodium falciparum *msp1 block 2 and msp2 allele prevalence and of infection complexity in two neighbouring Senegalese villages with different transmission conditions Trans R Soc Trop Med Hyg 1999 93 21 28 10450422 10.1016/s0035-9203(99)90323-1

[B13] Branch OH Takala S Kariuki S Nahlen BL Kolczak M Hawley W Lal AA *Plasmodium falciparum *genotypes, low complexity of infection, and resistance to subsequent malaria in participants in the Asembo Bay Cohort Project Infect Immun 2001 69 7783 7792 10.1128/IAI.69.12.7783-7792.2001 11705960 PMC98874

[B14] Polley SD Tetteh KK Cavanagh DR Pearce RJ Lloyd JM Bojang KA Okenu DM Greenwood BM McBride JS Conway DJ Repeat sequences in block 2 of *Plasmodium falciparu *merozoite surface protein 1 are targets of antibodies associated with protection from malaria Infect Immun 2003 71 1833 1842 10.1128/IAI.71.4.1833-1842.2003 12654798 PMC152097

[B15] Cavanagh DR Dodoo D Hviid L Kurtzhals JA Theander TG Akanmori BD Polley S Conway DJ Koram K McBride JS Antibodies to the N-terminal block 2 of *Plasmodium falciparu *merozoite surface protein 1 are associated with protection against clinical malaria Infect Immun 2004 72 6492 6502 10.1128/IAI.72.11.6492-6502.2004 15501780 PMC522997

[B16] Sakihama N Matsuo T Mitamura T Horii T Kimura M Kawabata M Tanabe K Relative frequencies of polymorphisms of variation in Block 2 repeats and 5' recombinant types of *Plasmodium falciparum *msp1 alleles Parasitol Int 2004 53 59 67 10.1016/j.parint.2003.11.002 14984836

[B17] Bharti PK Alam MT Boxer R Shukla MM Gautam SP Sharma YD Singh N Therapeutic efficacy of chloroquine and sequence variation in pfcrt gene among patients with falciparum malaria in central India Trop Med Int Health 2010 15 33 40 19912592 10.1111/j.1365-3156.2009.02425.x

[B18] Singh J Bhattacharyaji LM Rapid staining of malarial parasites by a water soluble stain Ind Med Gaz 1994 79 102 104 PMC5155780 29012018

[B19] Conway DJ Roper C Oduola AM Arnot DE Kremsner PG Grobusch MP Curtis CF Greenwood BM High recombination rate in natural populations of *Plasmodium falciparum* Proc Natl Acad Sci USA 1999 96 4506 4511 10.1073/pnas.96.8.4506 10200292 PMC16362

[B20] Conway DJ Rosario V Oduola AM Salako LA Greenwood BM McBride JS *Plasmodium falciparum*: intragenic recombination and nonrandom associations between polymorphic domains of the precursor to the major merozoite surface antigens Exp Parasitol 1991 73 469 480 10.1016/0014-4894(91)90071-4 1720396

[B21] Mahajan RC Farooq U Dubey ML Malla N Genetic polymorphism in *Plasmodium falciparu *vaccine candidate antigens Indian J Pathol Microbiol 2005 48 429 438 16366089

[B22] Ranjit MR Sharma YD Genetic polymorphism of falciparum malaria vaccine candidate antigen genes among field isolates in India AmJTrop Med Hyg 1999 61 103 108 10.4269/ajtmh.1999.61.103 10432065

[B23] Raj DK Das BR Dash AP Supakar PC Genetic diversity in the merozoite surface protein 1 gene of *Plasmodium falciparu *in different malaria-endemic localities AmJTrop Med Hyg 2004 71 285 289 15381807

[B24] Mehrizi AA Zakeri S Salmanian AH Sanati MH Djadid ND *Plasmodium falciparum*: sequence analysis of the gene encoding the C-terminus region of the merozoite surface protein-1, a potential malaria vaccine antigen, in Iranian clinical isolates Exp Parasitol 2008 118 378 385 10.1016/j.exppara.2007.10.001 18053992

[B25] Gómez D Chaparro J Rubiano C Rojas MO Wasserman M Genetic diversity of *Plasmodium falciparu *field samples from an isolated Colombian village AmJTrop Med Hyg 2002 67 611 616 10.4269/ajtmh.2002.67.611 12518851

[B26] Escalante AA Cornejo OE Rojas A Udhayakumar V Lal AA Assessing the effect of natural selection in malaria parasites Trends Parasitol 2004 20 388 395 10.1016/j.pt.2004.06.002 15246323

[B27] Talisuna AO Langi P Bakyaita N Egwang T Mutabingwa TK Watkins W Van Marck E D'Alessandro U Intensity of malaria transmission, antimalarial-drug use and resistance in Uganda: what is the relationship between these three factors? Trans R Soc Trop Med Hyg 2002 96 310 317 10.1016/S0035-9203(02)90108-2 12174786

[B28] Farnert A Williams TN Mwangi TW Ehlin A Fegan G Macharia A Lowe BS Montgomery SM Marsh K Transmission-dependent tolerance to multiclonal *Plasmodium falciparu *infection J Infect Dis 2009 200 1166 1175 10.1086/605652 19702508 PMC2741682

[B29] Singh N Shukla MM Chand G Bharti PK Singh MP Shukla MK Mehra RK Sharma RK Dash AP Epidemic of *Plasmodium falciparu *malaria in Central India, an area where chloroquine has been replaced by artemisinin-based combination therapy Trans R Soc Trop Med Hyg 2011 105 133 139 10.1016/j.trstmh.2010.11.002 21292291

[B30] Paul RE Hackford I Brockman A Muller-Graf C Price R Luxemburger C White NJ Nosten F Day KP Transmission intensity and *Plasmodium falciparum *diversity on the northwestern border of Thailand AmJTrop Med Hyg 1998 58 195 203 10.4269/ajtmh.1998.58.195 9502604

