# Supplementary material for: Genetic diversity in the block 2 region of the merozoite surface protein-1 of Plasmodium falciparum in central India
Source: Malar J. 2012 Mar 22;11:78. doi: 10.1186/1475-2875-11-78 (PMC3324372; doi:10.1186/1475-2875-11-78)
Supplement: Additional file 1 — Figure S1. Amino acid sequence alignment of the K1 allelic types of Plasmodium falciparum msp1 gene from central India. Figure S2. Amino acid sequence alignment of the MAD20 allelic types of Plasmodium falciparum msp1 gene from central India. Figure S3. Amino acid sequence alignment of the RO33 allelic types of Plasmodium falciparum msp1 gene from central India. [file 1475-2875-11-78-S1.DOCX]

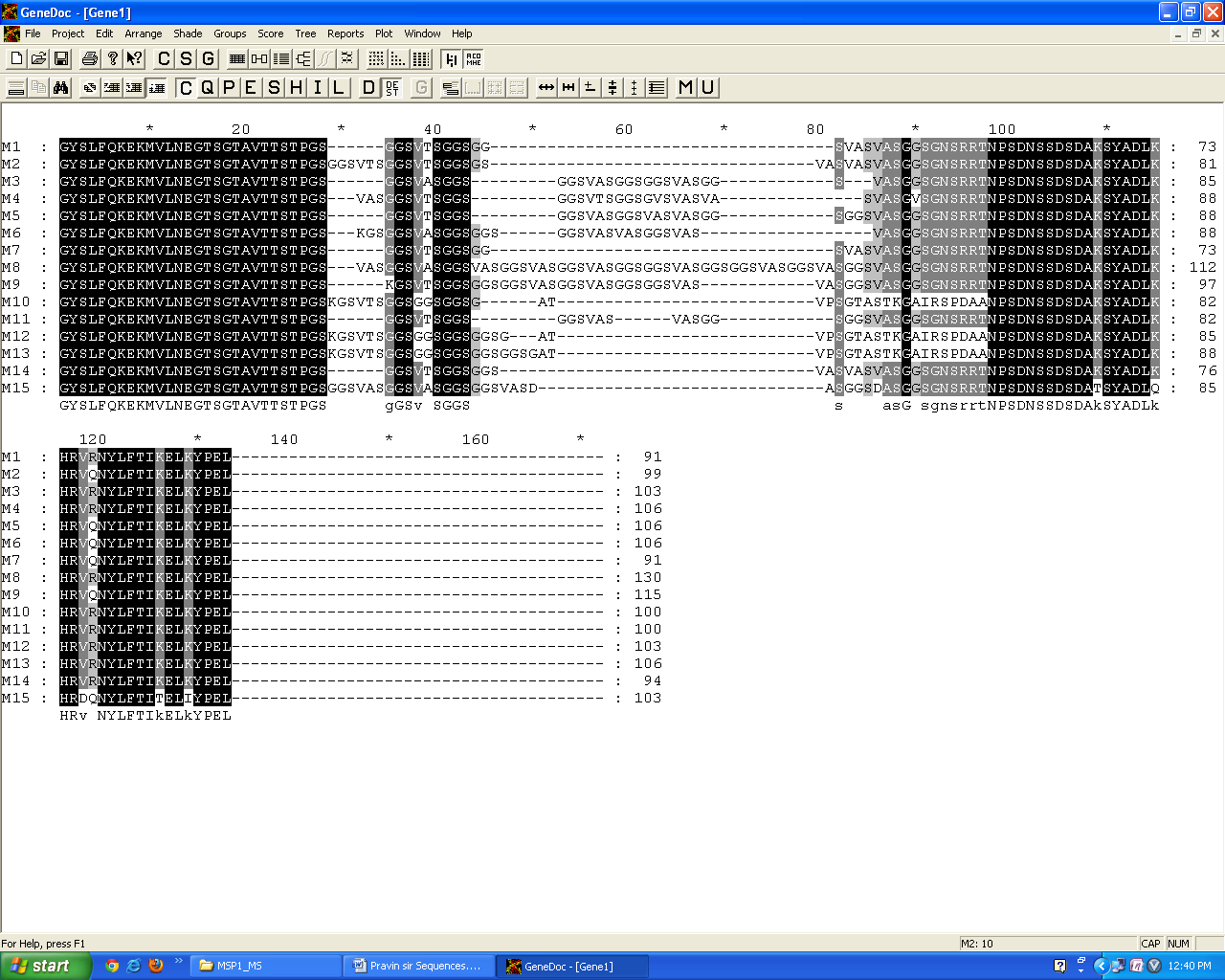


Additional figure1: Amino acid sequence alignment of the MAD20 allelic types of *Plasmodium falciparum msp1* gene from central India


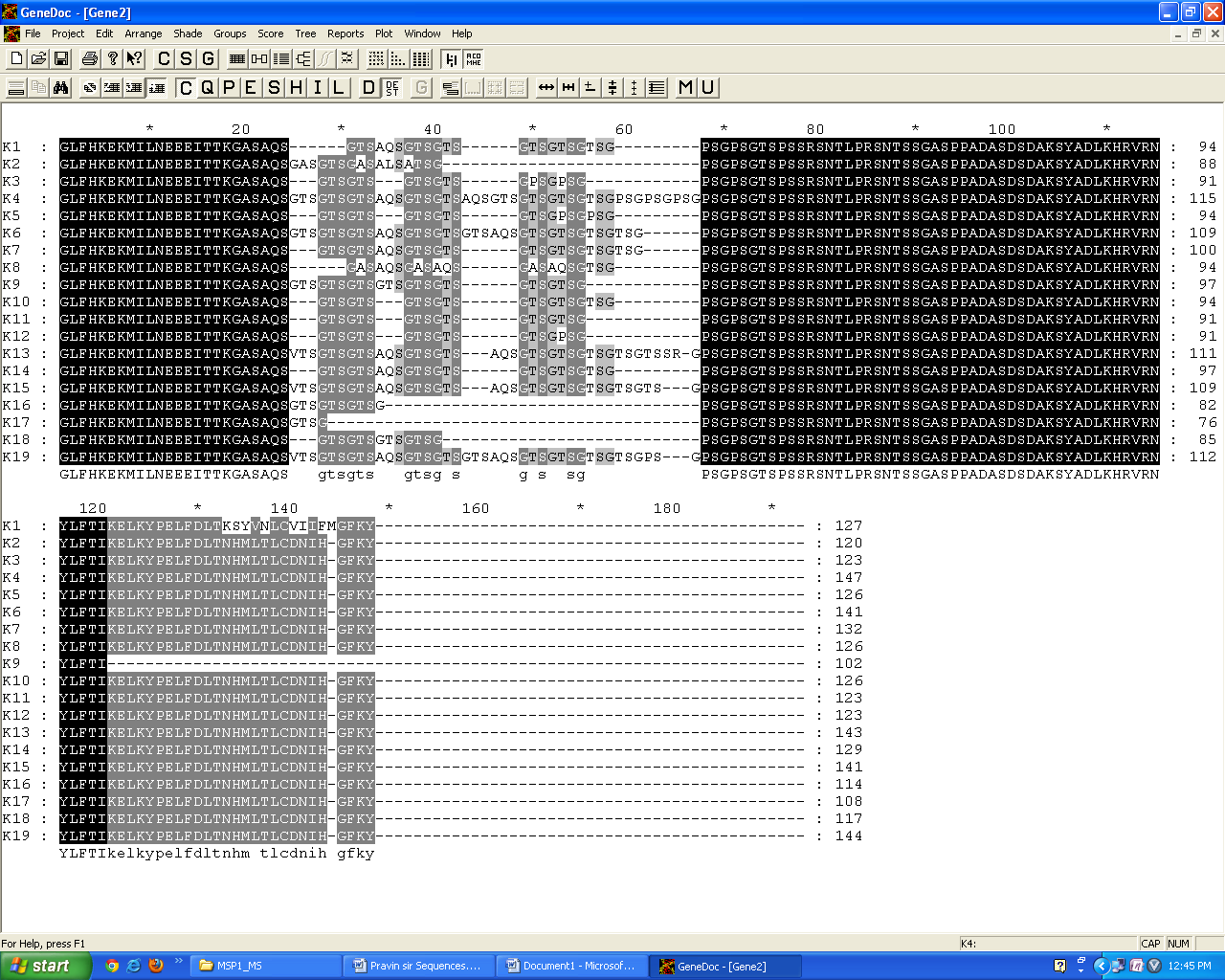


Additional figure2: Amino acid sequence alignment of the K1 allelic types of *Plasmodium falciparum msp1* gene from central India


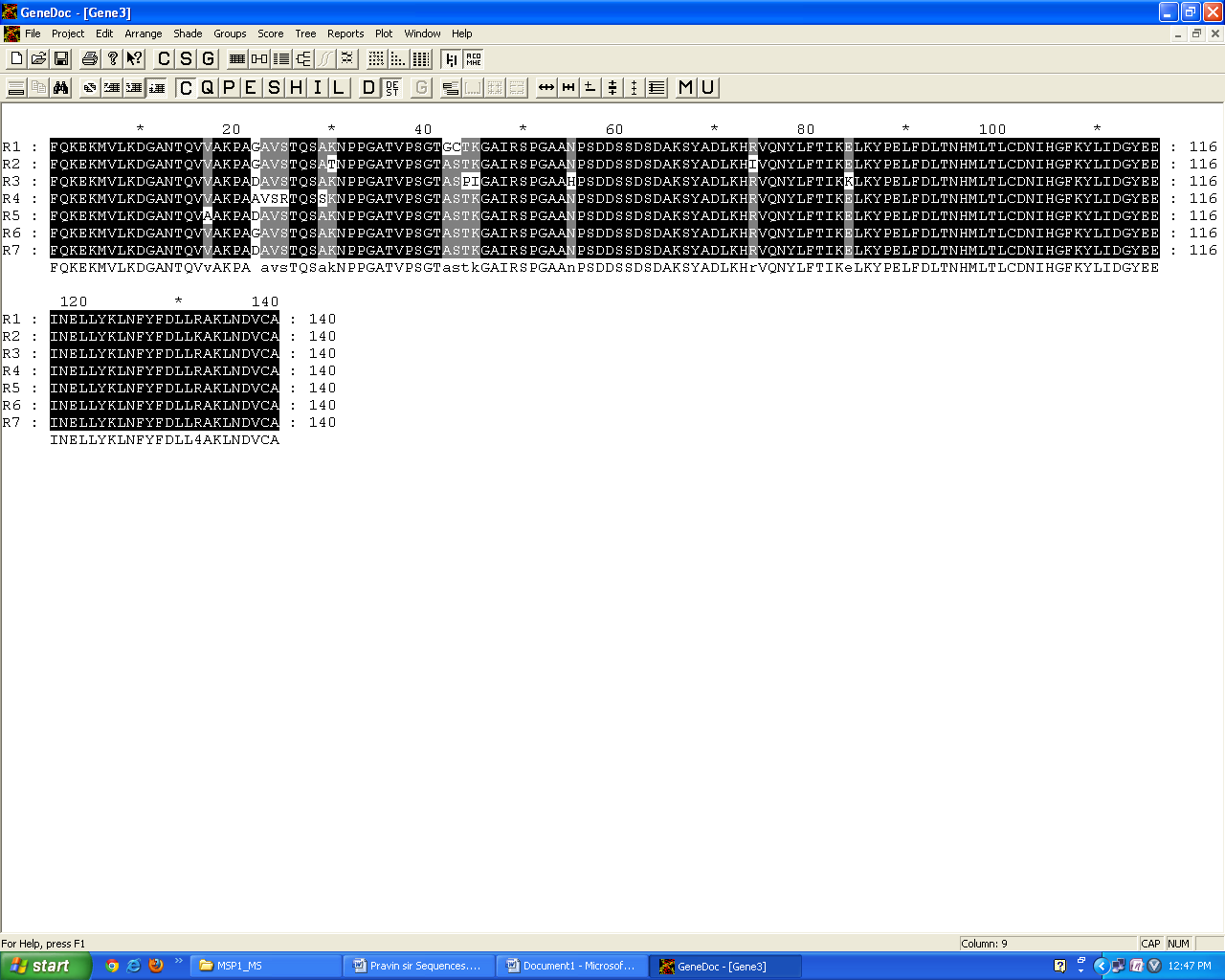


Additional figure3: Amino acid sequence alignment of the RO33 allelic types of *Plasmodium falciparum msp1* gene from central India
